# Perception to Dementia among final year medical students in a Singapore hospital: A prelude to Dementia Care planning

**DOI:** 10.1002/alz70858_097779

**Published:** 2025-12-24

**Authors:** Peng Soon Yoon, Kiat Sern Goh, Samuel TH Chew

**Affiliations:** ^1^ Changi General Hospital, Singapore, Singapore; ^2^ Changi General Hospital, Singapore, Singapore, Singapore

## Abstract

**Background:**

As the population in Singapore is ageing, it is expected that there will be more patients with dementia in the elderly, thus increasing the care burden among healthcare workers. To alleviate the burden of care especially for the inpatients with dementia, more training is done now emphasizing on person centered care with non‐pharmacological methods. The objective of this study is to know the perception and attitude to dementia among final year medical students who had undergone a three‐week student internship program (SIP) in the Department of Geriatric Medicine, Changi General Hospital, Singapore prior to further education and training on dementia care planning for future doctors in the department.

**Method:**

A self‐administered anonymous questionnaire was filled up by each final year medical student during the three‐week program when the student was in the department. Throughout the academic year, a total of 62 students completed the program in various batches. The questionnaire consisted of questions on their prior experience in dementia education, their perception on how important dementia was, their most concerning issue amongst people with dementia, and what was their preferred mode of learning. There was also one free text question on the reason if the participant chose ‘Very Important’.

**Result:**

Most students had some exposure to dementia prior to their Geriatric Medicine posting, with 43 had during their psychiatric medicine posting. 50 students rated dementia as ‘very important’ and the main reasons were “very common now” and “aging population”. Dementia issues concerning family members or caregivers rated highly were behavior issues, and poor safety awareness leading to fall risk. Regarding their preferred mode of learning, majority of the students chose tutorial (physical).

**Conclusion:**

There is strong awareness amongst medical students on the importance of dementia education and its challenges, and they were enthusiastic to learn more on dementia care.

